# Anxiety and depressive symptoms in patients with acute ischaemic stroke and cognitive dysfunction: a retrospective single-center study

**DOI:** 10.25122/jml-2026-0125

**Published:** 2026-08

**Authors:** Irina Maria Vlad, Dafin Fior Mureșanu, Lăcrămioara Perju-Dumbravă

**Affiliations:** 1 Doctoral School, Iuliu Hațieganu University of Medicine and Pharmacy Cluj-Napoca, Romania; 2 Department of Neurosciences, Iuliu Hațieganu University of Medicine and Pharmacy, Cluj-Napoca, Romania; 3 Neurology Clinic, Cluj County Emergency Clinical Hospital, Cluj-Napoca, Romania; 4 RoNeuro Institute for Neurological Research and Diagnostic, Cluj-Napoca, Romania

**Keywords:** acute ischemic stroke, cognitive dysfunction, anxiety, depression, neuropsychological assessment

## Abstract

Anxiety and depressive symptoms may coexist with cognitive dysfunction after stroke. This study characterized their frequency and severity during acute stroke hospitalization in patients with documented cognitive dysfunction. We conducted a retrospective, single-center study of 95 adults with acute ischaemic stroke (AIS) and cognitive dysfunction in Romania during 2024–2025. Anxiety and depressive symptoms were derived from neuropsychological conclusions and classified as absent/minimal, mild, moderate, or severe. In the main analysis, “elements” and the depression-specific “tendency” were classified as absent/minimal, while hybrid descriptors were assigned to the adjacent higher category. Sensitivity analyses examined alternative allocations. Associations were assessed using the chi-square, Fisher’s exact, or Kruskal–Wallis test. Anxiety severity was available for 94 patients and was distributed as follows: 26.6% absent/minimal, 16.0% mild, 50.0% moderate, and 7.4% severe. The severe category derived from “moderate–severe” descriptors. Overall, 73.4% had anxiety of at least mild severity. Depression severity was available for 86 patients, distributed as 74.4% absent/minimal, 17.4% mild, 7.0% moderate, and 1.2% severe; 25.6% had depression of at least mild severity. Under alternative low-level classifications, the anxiety–depression difference at the at-least-mild threshold narrowed from 47.8 to 28.1 and 9.0 percentage points. Anxiety remained more frequent. Sex was associated with both severity distributions. These proportions reflect documented severity rather than prevalence estimates derived from a uniform standardized instrument. Anxiety was more frequently documented and more often reached moderate-or-greater severity than depression. Routine neuropsychological conclusions may provide information on the affective profile during this phase of care. Longitudinal studies should examine affective, cognitive, and functional trajectories during recovery.

## Introduction

Cognitive impairment in patients with acute ischaemic stroke (AIS) can reflect multiple influences, including the stroke itself, stroke-related complications, pre-existing cognitive decline, and potentially reversible factors [1]. Post-stroke cognitive impairment encompasses the spectrum of cognitive impairment observed after an acute stroke, irrespective of the causal factor [1–3]. It ranges from milder deficits to post-stroke dementia and has implications for post-stroke functioning, independence, quality of life, rehabilitation, and institutionalization [1,3]. Its clinical expression is heterogeneous and may involve several domains simultaneously. Early identification of cognitive impairment is therefore an important component of post-stroke assessment and rehabilitation planning [1,3].

Anxiety and depressive symptoms are also frequent after stroke [4]. An updated systematic review and meta-analysis of 97 studies comprising 22 262 participants estimated post-stroke anxiety in 18.7% of patients when assessed by interview and in 24.2% when assessed using rating scales [5]. Post-stroke depression is similarly frequent: a systematic review and meta-analysis of 77 studies estimated its overall prevalence at 27%, with estimates of 24% using clinical interviews and 29% using rating scales [6]. Both reviews also showed variation by assessment method and study characteristics [5,6].

The relationship between anxiety, depression and cognitive dysfunction is particularly relevant. Post-stroke anxiety and depression arise through interacting biological, cognitive, behavioral, and social-environmental mechanisms [7]. These considerations are particularly relevant to the present study, as all included patients had documented cognitive dysfunction.

Anxiety, depression, and cognitive status may all change after stroke. In a study by Ebaid *et al*. of patients with mild ischemic stroke, cognitive performance and mood symptoms differed from those of stroke-free controls at both 3 and 12 months. Executive function improved significantly over time, while no association was found between mood scores and cognitive performance [8]. A longer-term study demonstrated heterogeneity in depressive symptom courses, with Ayis *et al*. identifying four trajectories over 5 years of follow-up [9]. These findings indicate that anxiety, depression, and cognitive status may change after stroke, supporting interpretation of the acute hospital assessment as a clinical reference point rather than a fixed description of subsequent recovery.

Against this background, the clinical expression of anxiety, depression, and cognitive impairment may differ according to the population studied and the stage of post-stroke recovery [1–8]. A previous Romanian study examined cognitive and affective (anxiety and depression) manifestations during post-stroke rehabilitation and found cognitive impairment to be weakly related to them, in contrast to the relationship between anxiety and depression [10].

The primary aim was to characterize the frequency and severity distribution of clinically documented anxiety and depressive symptoms during the index AIS hospitalization in patients with documented cognitive dysfunction. Among patients with both affective domains available, their coexistence and relative severity were additionally described, while exploratory secondary analyses examined differences according to age, sex, and aphasia type. The study was designed to characterize the acute affective profile documented during hospitalization rather than to estimate population-level prevalence or determine the onset, causes, or subsequent evolution of affective and cognitive manifestations. This acute profile was therefore considered a clinical reference point within potentially evolving affective, cognitive, and functional trajectories during post-stroke recovery.

## Material and Methods

### Study design and setting

Using routinely recorded hospital data, we performed a retrospective single-center study of adults admitted with AIS to Cluj County Emergency Clinical Hospital, Cluj-Napoca, Romania. The analysis was cross-sectional and was restricted to information recorded during the qualifying hospitalization in 2024–2025.

### Study population and eligibility criteria

Potentially eligible patients were identified among adults aged ≥18 years discharged with AIS as the principal diagnosis. Electronic case identification used the International Classification of Diseases, 10^th^ Revision (ICD-10) codes I63.3, I63.4, and I63.5 [11], after which the diagnosis was verified from the available clinical and/or neuroimaging documentation. These codes were selected because they represent the cerebral infarction codes routinely used for AIS at the study center. Eligibility additionally required cognitive dysfunction to be recorded as a secondary discharge diagnosis during the same admission and a neuropsychological assessment with sufficient information to determine anxiety and/or depressive symptoms.

The ICD-10 codes were used only for case identification. Vascular territory was determined separately from the clinical and neuroimaging documentation and classified as anterior circulation, posterior circulation, or multiple territories when more than one vascular territory was involved.

The presence of cognitive dysfunction was an eligibility criterion and defined the study population. Neither its severity nor the specific methods used to establish cognitive status were evaluated. Pre-existing cognitive dysfunction or dementia did not preclude inclusion.

Records were excluded if the review did not confirm AIS according to the study definition, if no neuropsychological assessment was available, if the available assessment was insufficient to determine either the anxiety or the depression domain, or if neurological or clinical severity prevented an interpretable neuropsychological assessment.

For patients with more than one qualifying admission during the study period, the earliest qualifying admission was retained, irrespective of whether it represented the patient’s first-ever stroke. All eligible cases identified during the study period were included.

### Data source and data collection

Study variables were abstracted retrospectively from the electronic health record. These included age, sex, vascular territory, previous ischemic stroke, previous cognitive dysfunction, previous psychiatric disorder, major comorbidities, stroke-related complications, documented alcohol use, and selected neurological and clinical findings.

For descriptive demographic and clinical variables, a condition not recorded in the available documentation was treated as absent. This rule did not apply to the anxiety and depression domains, for which insufficient documentation was treated as missing.

Association analyses were limited to age, sex, and aphasia type. The remaining clinical variables were used to characterize the cohort.

### Ascertainment of anxiety and depressive symptoms

Anxiety and depressive symptoms were identified from the final clinical conclusions of neuropsychological assessments performed during the index hospitalization by psychologists within the Department of Neurology. The analysis relied on these routine-care clinical interpretations rather than on retrospective reconstruction or comparison of individual psychometric instruments or numerical scores.

Standardized tests may have contributed to the assessment of individual patients, but they were not applied or documented uniformly across the cohort, and comparison of specific assessment methodologies was not an objective of the study. The final neuropsychological conclusion was therefore used as the common source for characterizing the documented anxiety and depression profile.

Anxiety and depression were considered present only when explicitly documented and absent when their absence was explicitly stated. If an otherwise interpretable assessment did not provide sufficient information to determine a given anxiety or depression domain, that domain was treated as missing. For readability, the terms anxiety and depression are subsequently used to refer to these clinically documented symptom-based variables and their recorded severity.

### Classification of anxiety and depression severity

The original affective descriptors were identified directly from the free-text final neuropsychological conclusions and reflected the severity terminology recorded in routine clinical documentation. These included “no”, “elements”, “tendency” (for depression), “mild”, “mild–moderate”, “moderate”, “moderate–severe”, and “severe”. The complete distribution of the original descriptors and their allocation under the main and alternative classifications is provided in Supplementary Table S1.

Supplementary Material

For the main analysis, the original descriptors were mapped to four predefined, mutually exclusive study severity categories: absent/minimal, mild, moderate, and severe. The absent/minimal category included explicit absence together with the low-level descriptors “elements” and “tendency”, considered to indicate symptoms below the clinical threshold of mild severity. Accordingly, “elements” was classified as absent/minimal for both affective domains, and the depression-specific descriptor “tendency” was also classified as absent/minimal. Original “mild”, “moderate”, and “severe” descriptors were assigned directly to the corresponding analytical categories. The hybrid descriptor “mild–moderate” was classified as moderate and “moderate–severe” as severe.

A separate sensitivity analysis examined the effect of assigning hybrid descriptors to the adjacent lower rather than higher severity category, so that “mild–moderate” was classified as mild and “moderate–severe” as moderate. This analysis assessed whether the overall severity distributions and the principal contrast between anxiety and depression changed materially according to the allocation of the hybrid descriptors.

Because the low-level descriptors “elements” and “tendency” lay immediately below the threshold of at least mild severity in the main classification, two additional sensitivity analyses examined alternative allocations of these descriptors. In the first low-level sensitivity analysis, “elements” retained its main classification as absent/minimal, while the depression-specific descriptor “tendency” was classified as mild. In the second low-level sensitivity analysis, “elements” was classified as mild for both affective domains and “tendency” as mild for depression. All other descriptors retained their main-analysis classification. These analyses were used to characterize how the estimated at-least-mild frequencies and the magnitude of the between-domain difference changed under alternative classifications.

The same four-category analytical framework (absent/minimal, mild, moderate, and severe) was used throughout the statistical analyses.

For descriptive purposes, the mild, moderate, and severe categories were grouped as symptoms of at least mild severity, representing the analytical threshold between descriptors classified below mild severity and symptoms reaching at least mild documented severity. Frequencies at this threshold and the corresponding anxiety–depression difference were examined under the main classification and the two alternative classifications of the low-level descriptors.

### Joint anxiety–depression severity pattern

Among patients with severity data available for both anxiety and depression, the two affective domains were examined at the individual level. Patients were descriptively classified according to whether anxiety severity exceeded depression severity, the two domains had equal severity, or depression severity exceeded anxiety severity.

Co-occurrence was additionally described using the threshold of at least mild severity, classifying patients as having neither affective domain at this level, anxiety only, depression only, or both anxiety and depression. Among patients in whom both domains reached at least mild severity, their relative severity was also described.

These analyses were descriptive, and no additional inferential comparisons were performed.

### Outcomes

The primary outcomes were the frequency and severity distributions of anxiety and depression, classified as absent/minimal, mild, moderate, or severe. The individual anxiety–depression severity pattern was examined descriptively among patients with both domains available.

Exploratory secondary analyses examined whether anxiety and depression severity distributions differed according to age, sex, and aphasia type. Sensitivity analyses assessed alternative classifications of hybrid and low-level descriptors and their effects on severity distributions and at-least-mild frequencies.

### Statistical analysis

Because all eligible cases identified during the study period were included, no a priori sample-size calculation was performed. Categorical data were summarised as *n* (%) and continuous data as median (interquartile range [IQR]). The chi-square or Fisher’s exact test was used for categorical comparisons, as appropriate, and the Kruskal–Wallis test was used for comparisons of age across severity categories. Tests were two-sided, with *P* < 0.05 considered statistically significant. Analyses according to age, sex, and aphasia type were exploratory and were not adjusted for multiple comparisons.

Analyses were performed using R version 4.3.2 (R Foundation for Statistical Computing, Vienna, Austria) [12] and Microsoft Excel (Microsoft Corporation, Redmond, WA, USA).

### Ethical considerations

The study was approved by the Ethics Committee of the Iuliu Hațieganu University of Medicine and Pharmacy Cluj-Napoca (approval no. 165/17 June 2026) and the Ethics Committee of Cluj County Emergency Clinical Hospital (approval no. 18622/28 May 2026). The study was conducted in accordance with the Declaration of Helsinki and the General Data Protection Regulation (GDPR). The requirement for informed consent was waived because of the retrospective design of the study.

## Results

### Study population

A total of 95 patients met the eligibility criteria and constituted the final study population. The median age was 74 years (IQR 69.5–78.0), 83.2% were aged ≥65 years, and 40.0% were men. Most strokes involved the anterior circulation (69.5%). Additional demographic and clinical characteristics are summarised in Table 1.

**Table 1:** Demographic and clinical characteristics of the study population (*n* = 95)

Characteristic	*n*	%
**Demographic characteristics**		
Age (years), median (IQR)	74 (69.5–78.0)	—
Age ≥65 years	79	83.2
Female sex	57	60.0
**Vascular territory**		
Anterior territory	66	69.5
Multiple territories	2	2.1
Posterior territory	27	28.4
**Medical history**		
Previous ischaemic stroke	33	34.7
Previous cognitive dysfunction	3	3.2
Previous psychiatric disorder	5	5.3
**Comorbidities**		
Hypertension	88	92.6
Atrial fibrillation	18	18.9
Coronary artery disease	20	21.1
Heart failure	17	17.9
Dyslipidaemia	50	52.6
Type 2 diabetes mellitus	35	36.8
Chronic kidney disease	10	10.5
Chronic respiratory disease	8	8.4
Hypothyroidism	16	16.8
Current or previous malignancy	12	12.6
Chronic liver disease	11	11.6
**Stroke-related and clinical findings**		
Major stroke-related complications	28	29.5
Documented alcohol use	10	10.5
Any aphasia	15	15.8
Dysarthria	23	24.2
Hearing impairment	13	13.7
Dysphagia	4	4.2
Motor deficit	57	60.0

Data are presented as *n* (%) for categorical variables and as median (IQR) for age; percentages were calculated with the total study population (*n* = 95) as the denominator. Conditions not recorded in the available documentation were treated as absent. IQR, interquartile range; *n*, number of patients.

### Distribution of documented anxiety and depression severity

Anxiety severity was available for 94 patients. In the main analysis, anxiety was classified as absent/minimal in 25 patients (26.6%), mild in 15 (16.0%), moderate in 47 (50.0%), and severe in 7 (7.4%). Overall, 69 of 94 patients (73.4%) had anxiety of at least mild documented severity.

Depression severity was available for 86 patients. Depression was classified as absent/minimal in 64 patients (74.4%), mild in 15 (17.4%), moderate in 6 (7.0%), and severe in 1 (1.2%). Depression of at least mild documented severity was therefore present in 22 of 86 patients (25.6%).

The two affective domains showed markedly different severity distributions: anxiety was most commonly moderate, whereas depression was predominantly absent/minimal (Table 2).

**Table 2:** Distribution of documented anxiety and depression severity

Severity category	Anxiety (*n* = 94)		Depression (*n* = 86)	
	Main analysis	Hybrid sensitivity analysis	Main analysis	Hybrid sensitivity analysis
Absent/minimal	25 (26.6)	25 (26.6)	64 (74.4)	64 (74.4)
Mild	15 (16.0)	19 (20.2)	15 (17.4)	18 (20.9)
Moderate	47 (50.0)	50 (53.2)	6 (7.0)	3 (3.5)
Severe	7 (7.4)	0 (0.0)	1 (1.2)	1 (1.2)

Data are presented as *n* (%). In the main analysis, the hybrid descriptors “mild–moderate” and “moderate–severe” were assigned to the adjacent higher category; in the hybrid sensitivity analysis, they were assigned to the adjacent lower category. *n*, number of patients.

Reassignment of the hybrid descriptors changed the distribution between adjacent severity categories but did not alter the proportion of patients classified as having symptoms of at least mild severity. Moderate-or-greater anxiety was documented in 57.4% of patients in the main analysis and in 53.2% under the alternative hybrid allocation, compared with 8.1% and 4.7%, respectively, for depression.

The principal effect concerned the severe anxiety category. None of the original neuropsychological conclusions used the descriptor “severe” for anxiety. All seven patients classified as severe in the main analysis had originally been documented as having “moderate–severe” anxiety. When “moderate–severe” was instead assigned to the adjacent lower category, the severe anxiety category decreased from 7.4% to 0%. For depression, no assessment used the descriptor “moderate–severe”, and the single original “severe” descriptor therefore remained severe under both classifications. Reassignment of the three “mild–moderate” cases from moderate to mild reduced the moderate category from 7.0% to 3.5% and increased the mild category from 17.4% to 20.9%.

### Anxiety–depression severity pattern in patients with both domains available

Both anxiety and depression severity were available for 85 patients. Under the main classification, anxiety severity exceeded depression severity in 58 patients (68.2%), while the two domains had equal documented severity in 27 (31.8%). Depression severity did not exceed anxiety severity in any patient (Table 3). Among the 27 patients with equal severity, 20 had both affective domains classified as absent/minimal, and seven had equal severity at a level of at least mild.

**Table 3:** Anxiety–depression severity pattern in patients with both domains available (*n* = 85)

Anxiety severity	Depression severity				Total
	Absent/minimal	Mild	Moderate	Severe	
Absent/minimal	20	0	0	0	20
Mild	10	2	0	0	12
Moderate	32	10	4	0	46
Severe	1	3	2	1	7
Total	63	15	6	1	85

When co-occurrence was considered using the descriptive threshold of at least mild severity, 43 of 85 patients (50.6%) had anxiety of at least mild severity without depression reaching that level, 22 (25.9%) had both anxiety and depression of at least mild severity, and 20 (23.5%) had neither domain at this level. No patient had depression of at least mild severity without anxiety also reaching this threshold. Among the 22 patients with both affective domains at least mild, anxiety severity exceeded depression severity in 15 and was equal in seven.

### Sensitivity of the at-least-mild threshold to low-level descriptors

The sensitivity of the at-least-mild threshold reflected the frequency of the low-level descriptors used in the original neuropsychological conclusions. “Elements” was documented in 16 of 94 anxiety assessments (17.0%) and in 31 of 86 depression assessments (36.0%), while the depression-specific descriptor “tendency” was documented in 17 of 86 assessments (19.8%). Their complete distribution and analytical allocation are shown in Supplementary Table S1.

Under the main classification, anxiety of at least mild severity was documented in 69 of 94 patients (73.4%) and depression in 22 of 86 (25.6%), a difference of 47.8 percentage points. When the depression-specific descriptor “tendency” was classified as mild, the difference narrowed to 28.1 percentage points (73.4% vs 45.3%), and when “elements” was additionally classified as mild for both domains, it narrowed further to 9.0 percentage points (90.4% vs 81.4%) (Table 4). Anxiety remained more frequent under all three classifications. These low-level reclassifications did not affect the proportions with moderate-or-greater documented severity.

**Table 4:** At-least-mild anxiety and depression under alternative classifications of low-level descriptors

Classification	Anxiety ≥mild, *n*/*N* (%)	Depression ≥mild, *n*/*N* (%)	Difference in proportions (percentage points)†
Main classification	69/94 (73.4)	22/86 (25.6)	47.8
Low-level sensitivity 1: “tendency” → mild	69/94 (73.4)	39/86 (45.3)	28.1
Low-level sensitivity 2: “elements” and “tendency” → mild	85/94 (90.4)	70/86 (81.4)	9.0

In the main classification, “elements” and the depression-specific descriptor “tendency” were classified as absent/minimal; the low-level sensitivity analyses reclassified only the descriptors specified in the table. †Difference calculated as the anxiety percentage minus the depression percentage, using domain-specific denominators. *n*, number of patients at or above mild severity; *N*, number of patients with the domain available.

### Exploratory comparisons according to age, sex, and aphasia type

Anxiety severity differed according to sex (*P* < 0.001); anxiety of at least mild severity was documented in 53 of 57 women (93.0%) and 16 of 37 men (43.2%). Depression severity also differed according to sex (*P* = 0.018), with depression of at least mild severity documented in 18 of 52 women (34.6%) and 4 of 34 men (11.8%).

Age did not differ across anxiety or depression severity categories (*P* = 0.954 and *P* = 0.838, respectively), and no statistically detectable differences were observed according to aphasia type (*P* = 0.917 and *P* = 1.000, respectively) (Supplementary Table S2).

These exploratory comparisons should be interpreted cautiously because some severity categories, particularly moderate and severe depression, contained few patients.

## Discussion

### Principal findings

This study characterized the frequency and documented severity of anxiety and depressive symptoms during the index AIS hospitalization in patients with cognitive dysfunction.

Three findings are particularly relevant. First, the two affective domains showed markedly different documented severity distributions. Under the main classification, in which “elements” and “tendency” were considered below the clinical threshold of mild severity, anxiety of at least mild severity was documented in 73.4% of patients with available anxiety data and depression in 25.6%, a difference of 47.8 percentage points. However, the magnitude of this difference changed substantially under the alternative classifications of the low-level descriptors: it narrowed from 47.8 percentage points in the main analysis to 28.1 percentage points when the depression-specific descriptor “tendency” was classified as mild and to 9.0 percentage points when “elements” was additionally classified as mild for both domains. Anxiety nevertheless remained more frequent at the at-least-mild threshold under all three classifications.

The distinction at higher levels of documented severity was more stable: moderate-or-greater anxiety was documented in 57.4% of patients under the main allocation and 53.2% under the hybrid-sensitivity allocation, compared with 8.1% and 4.7%, respectively, for depression. The severe anxiety category requires specific interpretation: no patient was originally described as having severe anxiety. All seven patients classified as severe in the main analysis had an original descriptor of “moderate–severe”. When these cases were allocated to the adjacent lower category, severe anxiety decreased from 7.4% to 0%. The difference between the two affective domains is therefore more appropriately characterized by their overall severity distributions, particularly the substantially greater representation of anxiety at moderate or greater severity, than by the binary at-least-mild threshold alone.

Second, among patients with both affective domains available, the joint distribution provided a descriptive view of how anxiety and depression were recorded for individual patients. Under the main classification, anxiety severity exceeded depression severity in 58 of 85 patients (68.2%), was equal in 27 (31.8%), and was lower in none. All patients with depression of at least mild severity also had anxiety at that level. These findings should be interpreted in the context of the considerably different severity distributions and of the classification used for the low-level descriptors, and should not be considered evidence of a distinct affective construct independent of those distributions.

Third, sex was the demographic characteristic associated with affective severity in the exploratory analyses, with higher documented anxiety and depression severity among women. These comparisons were unadjusted and should not be interpreted as evidence of a direct effect of sex.

Taken together, the findings indicate that the clearest distinction between anxiety and depression in this cohort concerned documented severity, with anxiety substantially more often recorded at moderate or greater severity. At the at-least-mild threshold, the magnitude of the difference depended considerably on how low-level clinical terminology was operationalized.

Previous acute and early post-stroke studies have also reported anxiety more frequently than depressive symptoms. Fure *et al*. observed anxiety in 26.4% and depressive symptoms in 14.0% of patients assessed 3–7 days after stroke-unit admission for AIS, with anxiety additionally associated with lower Mini-Mental State Examination scores [13]. More recently, anxiety was also reported more frequently than depression during early follow-up after AIS treated with mechanical thrombectomy [14]. However, the anxiety frequency observed in the present study was substantially greater than that reported in these studies and than the pooled post-stroke anxiety estimates by Knapp *et al*. and Liu *et al*. Under the main classification, the depression frequency of 25.6% was numerically close to the pooled estimate of approximately 27% [6], whereas the anxiety frequency of 73.4% was considerably higher than the pooled estimates of 18.7% by interview and 24.2% by rating scale [5]. However, the characteristics of the cohort and the selection criteria limit direct comparisons.

The high frequency of documented anxiety should also be considered in the context of the acute stroke phase. In a study by Tan, illness uncertainty, acute stress responses, and anxiety were closely linked [15]. The present study was not designed to determine the causes of anxiety during hospitalization. These comparisons are contextual rather than formal because the populations, assessment approaches, clinical settings, and symptom-classification rules differed. The high anxiety frequency in the present cohort should therefore be interpreted in relation to both its clinically selected population and the characteristics of routine clinical documentation and classification.

### Anxiety and depression within the context of cognitive dysfunction

Anxiety and depressive symptoms are both well recognized after stroke but represent related rather than interchangeable affective dimensions [5,6]. Their frequency, severity, clinical expression, and subsequent course may differ.

The present findings illustrate this distinction. Anxiety was more prominent than depression across the main and alternative low-level classifications, although the magnitude of the difference at the at-least-mild threshold varied substantially. The difference was clearer at moderate or greater severity, where anxiety was consistently much more frequent.

The paired analysis provides a complementary within-patient description, showing that, under the main classification, anxiety severity more often exceeded depression severity in the same patients.

This distinction may be particularly relevant in the context of cognitive dysfunction. Post-stroke cognitive impairment can affect attention, executive functioning, and memory, with important implications for everyday functioning and recovery [1]. In another study, domain-specific cognitive impairments were associated with greater depressive symptom severity at 6 months [16].

The relationship may also operate in the opposite direction. Affective manifestations may influence cognitive performance or complicate its clinical interpretation after stroke. A recent American Heart Association/American Stroke Association scientific statement on cognitive impairment after stroke emphasizes considering mood when evaluating post-stroke cognition [1]. Furthermore, a 4-month prospective study showed that changes in depressive symptom severity may accompany changes in cognitive performance, including verbal fluency, among women but not men [17]. These observations support interpreting post-stroke cognitive dysfunction in the context of coexisting affective and other clinical factors.

The present study does not establish why anxiety was more prominent than depression during the acute admission. It does, however, show that the two domains were not expressed with equivalent documented severity, and it supports their separate clinical consideration.

### Sex and other clinical characteristics

Sex was associated with affective severity in the exploratory analyses. Under the main classification, anxiety of at least mild severity was documented in 93.0% of women compared with 43.2% of men, while depression of at least mild severity was documented in 34.6% and 11.8%, respectively. These comparisons were unadjusted and exploratory and should not be interpreted as evidence of a direct effect of sex.

Previous studies have also reported greater post-stroke anxiety among women, although the magnitude and age pattern differed substantially from those observed in the present cohort. In the community-based study by Broomfield *et al*., definite anxiety was present in 19.8% of women and 13.1% of men and decreased markedly with increasing age [18], whereas the present cohort was predominantly older and showed no detectable relationship between age and anxiety severity. These differences likely reflect important variation in population selection, clinical setting, timing after stroke, and assessment method; the studies are therefore not directly comparable.

Furthermore, no statistically detectable differences in anxiety or depression severity were observed according to age or aphasia type in the present exploratory analyses. Given the cohort size and the small numbers in some severity categories, these findings should not be interpreted as evidence that these characteristics have no relationship with affective manifestations generally.

### The acute affective profile as a reference point within the post-stroke trajectory

The affective findings in the present study characterize a defined acute phase: the index AIS hospitalization in patients with documented cognitive dysfunction. This profile should therefore not necessarily be interpreted as the onset of either affective or cognitive manifestations, nor as a fixed long-term state. Rather, it provides a clinically defined reference point within an evolving post-stroke course.

Longitudinal evidence supports this distinction. Depressive symptoms may change substantially during the first year after stroke, with almost half of patients in one recent cohort changing symptom status over time [19]. Anxiety also follows heterogeneous courses, with persistent, fluctuating, and absent symptom trajectories reported during rehabilitation and longer-term follow-up [20]. Cognitive status is similarly dynamic: longitudinal data identified distinct trajectories ranging from stable mild impairment to substantial improvement or decline, with much of the cognitive recovery occurring within the first 6 months, while some domain-specific deficits persisted longer [21].

These changing courses are clinically relevant because affective, cognitive, and functional recovery may be associated [22,23]. Early affective status may also be associated with recovery. In a longitudinal study by Li *et al*. of an AIS cohort, depressive symptoms documented during acute hospitalization were associated with less favorable functional recovery trajectories over 6 months [23].

Although the present study cannot establish comparable longitudinal relationships, these findings provide a rationale for examining whether the identified acute profile is associated with later cognitive and functional outcomes.

Clinically, the acute neuropsychological conclusion may therefore serve not as a definitive psychological characterization but as information to be reconsidered as recovery progresses. Although the findings are not population-level estimates, the prominent documentation of anxiety in this cohort supports particular attention to anxiety alongside depression during acute post-stroke affective assessment, particularly in patients with cognitive dysfunction.

Whereas the present study relied on routine neuropsychological conclusions, guidelines such as the Canadian stroke best practice recommendations recommend validated screening for post-stroke depression and indicate that screening for anxiety may also be considered [24]. In this retrospective study, the final neuropsychological conclusion provided a common source for characterizing the anxiety and depression profile documented during routine acute care, as individual instruments were not applied uniformly across the cohort.

Prospective studies following anxiety and depressive symptoms, cognition, and functional status from acute hospitalization through rehabilitation and longer-term recovery are needed to determine how these trajectories interact, whether the acute profile is associated with subsequent cognitive and functional recovery, and whether their evolution can inform more individualized post-stroke care.

### Strengths and limitations

Several strengths should be noted. The study characterized both the frequency and the documented severity of anxiety and depression using routine neuropsychological information from acute stroke care. The within-patient analysis additionally described the coexistence and relative severity of the two affective domains, while sensitivity analyses showed how alternative classification of the original clinical terminology affected the derived severity distributions and the anxiety–depression contrast.

Several limitations should also be considered. The retrospective single-center design, discharge-code-based case identification, and requirement for documented cognitive dysfunction and an interpretable neuropsychological assessment may have introduced selection bias and may limit generalisability. Patients with severe neurological or communication impairment may therefore have been under-represented.

A uniformly applied standardised affective instrument was not available across the cohort, and detailed assessment procedures could not be consistently reconstructed retrospectively. The analysis consequently relied on the final neuropsychological conclusions, with potential variability in assessment and documentation. The original clinical terminology also required analytical grouping into severity categories, including decisions regarding low-level and hybrid descriptors. Sensitivity analyses showed that allocation of the low-level descriptors influenced the magnitude of the anxiety–depression difference at the at-least-mild threshold. The reported frequencies should therefore be interpreted as clinically documented affective severity within this cohort rather than as prevalence estimates directly comparable with those derived from standardized instruments or structured diagnostic assessments. In addition, the timing of the neuropsychological examination was not available, which is a further limitation.

Depression data were unavailable for nine patients, and the small numbers with moderate or severe depression limited some exploratory comparisons.

## Conclusion

Among patients hospitalized with AIS and documented cognitive dysfunction, anxiety and depression showed distinct documented severity profiles during the index hospitalization. Anxiety was more frequently documented and more often reached moderate-or-greater severity than depression, while the magnitude of the difference at the at-least-mild threshold varied according to the classification of low-level clinical descriptors. The reported frequencies therefore reflect clinically documented severity rather than population-level prevalence.

Routine neuropsychological conclusions may provide clinically relevant information on the anxiety and depressive profile during this phase of care. Because anxiety, depression, cognitive status, and functional status may change during recovery, longitudinal studies are needed to examine how these trajectories evolve over time. Furthermore, the findings of this study support the post-stroke evaluation of anxiety and depression alongside cognition, given their potential relevance to cognitive performance, communication, rehabilitation participation, and functional recovery.

## Data Availability

Further data are available from the corresponding author upon reasonable request.
