# Supplementary material for: Anxiety and depressive symptoms in patients with acute ischaemic stroke and cognitive dysfunction: a retrospective single-center study
Source: J Med Life. 2026 Aug;19(8):618–26. doi: 10.25122/jml-2026-0125 (PMC13674036; doi:10.25122/jml-2026-0125)
Supplement: Supplementary file 1 [file JMedLife-19-618-s001.pdf]

Table S1. Distribution and analytical classification of originally documented anxiety and depression severity descriptors

Representative Romanian wording: anxiety	Representative Romanian wording: depression	English gloss	Frequency, *n* (%)		Analytical category			
			Anxiety (*n* = 94)	Depression (*n* = 86)	Main analysis	Hybrid-descriptor sensitivity	Low-level sensitivity 1†	Low-level sensitivity 2‡
„Fără anxietate”	„Fără depresie”	No	9 (9.6)	16 (18.6)	Absent/ minimal	Absent/ minimal	Absent/ minimal	Absent/ minimal
„Elemente de anxietate” / „elemente anxioase”	„Elemente de depresie” / „elemente depresive”	Elements	16 (17.0)	31 (36.0)	Absent/ minimal	Absent/ minimal	Absent/ minimal	Mild
—	„Tendințe depresive”	Tendency	—	17 (19.8)	Absent/ minimal (depression only)	Absent/ minimal (depression only)	Mild (depression only)	Mild (depression only)
„Anxietate ușoară” / „simptome de anxietate ușoară”	„Depresie ușoară” / „simptome depresive ușoare”	Mild	15 (16.0)	15 (17.4)	Mild	Mild	Mild	Mild
„Anxietate ușoară–moderată”	„Depresie ușoară–moderată”	Mild–moderate	4 (4.3)	3 (3.5)	Moderate	Mild	Moderate	Moderate
„Anxietate moderată”	„Depresie moderată”	Moderate	43 (45.7)	3 (3.5)	Moderate	Moderate	Moderate	Moderate
„Anxietate moderat–severă”	„Depresie moderat–severă”	Moderate–severe	7 (7.4)	0 (0.0)	Severe	Moderate	Severe	Severe
„Anxietate severă”	„Depresie severă”	Severe	0 (0.0)	1 (1.2)	Severe	Severe	Severe	Severe
Total available			94 (100.0)	86 (100.0)				
Missing			1	9				

Minor grammatical and lexical variants with the same clinical meaning and severity were consolidated; representative Romanian wording is shown. Percentages were calculated with the number of patients with the respective domain available as the denominator. Main analysis: “elements” and “tendency” were classified as absent/minimal, and the hybrid descriptors “mild–moderate” and “moderate–severe” were assigned to the adjacent higher category. Hybrid-descriptor sensitivity analysis: “mild–moderate” was assigned to mild and “moderate–severe” to moderate; all other descriptors retained their main-analysis classification. A dash (—) indicates that the descriptor was not applicable to that affective domain. †Low-level sensitivity 1: “tendency” was classified as mild for depression; “elements” remained absent/minimal in both domains; all other descriptors retained their main-analysis classification. ‡Low-level sensitivity 2: “elements” was classified as mild for both anxiety and depression, and “tendency” as mild for depression; all other descriptors retained their main-analysis classification. *n*, number of patients.

© 2026 by the authors. This article is an open access article distributed under the terms and conditions of the Creative Commons Attribution (CC BY 4.0) license.

Table S2. Selected demographic and clinical characteristics according to affective severity

**(A) Anxiety severity (*n* = 94)**	**Absent/minimal (*n* = 25)**	**Mild (*n* = 15)**	**Moderate (*n* = 47)**	**Severe (*n* = 7)**	***P***
Age (years), median (IQR)	73 (71–78)	73 (71–78)	74 (68.5–77)	78 (65–78)	0.954
Female sex, *n* (%)	4 (16.0)	12 (80.0)	36 (76.6)	5 (71.4)	<0.001
Aphasia type, *n* (%)					0.917
Mixed	1 (4.0)	0 (0.0)	1 (2.1)	0 (0.0)	
Motor	2 (8.0)	3 (20.0)	5 (10.6)	1 (14.3)	
Sensory	1 (4.0)	0 (0.0)	1 (2.1)	0 (0.0)	
None	21 (84.0)	12 (80.0)	40 (85.1)	6 (85.7)	
**(B) Depression severity (*n* = 86)**	**Absent/minimal (*n* = 64)**	**Mild (*n* = 15)**	**Moderate (*n* = 6)**	**Severe (*n* = 1)**	***P***
Age (years), median (IQR)	73 (70–78)	77 (65–78)	75.5 (68.75–77)	78 (78–78)	0.838
Female sex, *n* (%)	34 (53.1)	13 (86.7)	5 (83.3)	0 (0.0)	0.018
Aphasia type, *n* (%)					1.000
Mixed	2 (3.1)	0 (0.0)	0 (0.0)	0 (0.0)	
Motor	8 (12.5)	2 (13.3)	0 (0.0)	0 (0.0)	
Sensory	2 (3.1)	0 (0.0)	0 (0.0)	0 (0.0)	
None	52 (81.3)	13 (86.7)	6 (100.0)	1 (100.0)	

Data are presented as median (IQR) or *n* (%); percentages were calculated within each severity category. IQR, interquartile range; *n*, number of patients.

© 2026 by the authors. This article is an open access article distributed under the terms and conditions of the Creative Commons Attribution (CC BY 4.0) license.
